# Towards understanding the myometrial physiome: approaches for the construction of a virtual physiological uterus

**DOI:** 10.1186/1471-2393-7-S1-S3

**Published:** 2007-06-01

**Authors:** Michael John Taggart, Andrew Blanks, Sanjay Kharche, Arun Holden, Bin Wang, Henggui Zhang

**Affiliations:** 1 Maternal and Fetal Health Research Centre, University of Manchester, St Mary's Hospital, Hathersage Road, Manchester, M13 0JH, UK; 2 Clinical Sciences Research Centre, Warwick Medical School, Coventry, UK; 3 School of Physics, University of Manchester, Manchester, M13 9PL, UK; 4 Institute of Membrane & Systems Biology, University of Leeds, Leeds, UK; 5 School of Engineering and Physical Sciences, University of Aberdeen, Aberdeen, UK

## Abstract

Premature labour (PTL) is the single most significant factor contributing to neonatal morbidity in Europe with enormous attendant healthcare and social costs. Consequently, it remains a major challenge to alleviate the cause and impact of this condition. Our ability to improve the diagnosis and treatment of women most at risk of PTL is, however, actually hampered by an incomplete understanding of the ways in which the functions of the uterine myocyte are integrated to effect an appropriate biological response at the multicellular whole organ system. The level of organization required to co-ordinate labouring uterine contractile effort in time and space can be considered immense. There is a multitude of what might be considered mini-systems involved, each with their own regulatory feedback cycles, yet they each, in turn, will influence the behaviour of a related system. These include, but are not exclusive to, gestational-dependent regulation of transcription, translation, post-translational modifications, intracellular signaling dynamics, cell morphology, intercellular communication and tissue level morphology.

We propose that in order to comprehend how these mini-systems integrate to facilitate uterine contraction during labour (preterm or term) we must, in concert with biological experimentation, construct detailed mathematical descriptions of our findings. This serves three purposes: firstly, providing a quantitative description of series of complex observations; secondly, proferring a database platform that informs further testable experimentation; thirdly, advancing towards the establishment of a virtual physiological uterus and *in silico *clinical diagnosis and treatment of PTL.

## Background

*'The challenge to interpret this vast volume of data at the genomic and proteomic level in terms of function at higher levels is exactly what modern physiology is about.' *[1].

The development of improved methods of diagnosis and treatment of preterm labour (PTL) is the major task facing obstetric research in this new millennium. PTL accounts for 6–8% of all births across Europe (~6% in the UK) [2]. Of even greater significance is that 75% of neonatal deaths, and most neonatal intensive care admissions, arise from preterm babies born before 32 weeks of pregnancy [3]. Oft-overlooked is the realisation that the impact of PTL is long-lasting; prematurely born children face a high risk of disability and life-long ill health. In a recent follow-up study of 308 UK children born prematurely (<26 weeks), the rates of severe, moderate and mild disability at 6 years of age were 22%, 24% and 34% respectively [4]. The short- and long-term economic consequences of preterm birth also place a considerable burden on society as a whole [5]. It is, therefore, a considerable cause of concern that the prevention of PTL, or the treatment of established PTL, remains seriously inadequate; in fact, the rates of PTL have not declined in the last 20 years. It is paramount, therefore, that we advance our understanding of uterine physiology, in all its complexities, in order to improve our diagnosis and treatment of PTL.

**Figure 1:**
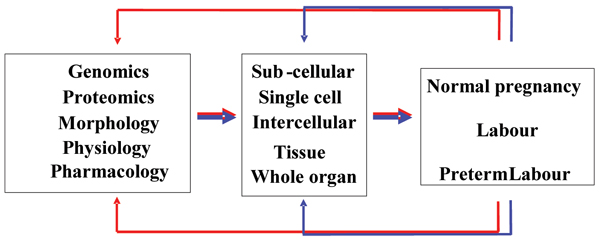
**Network domains for the myometrial physiome**. Interlinked modalities that one must consider in developing an understanding of the virtual physiological uterus.

Perhaps our greatest challenge ahead, as evoked in the quotation above, is to consider how we can assimilate the vast arrays of molecular and cellular information currently generated into a proper physiological context: biological systems are often dynamic, non-linear, non-homogeneous and operate at various mesmerising levels of organisation and hierarchy. Still yet more complexity is introduced with the remodelling of physiological events during pregnancy and labour. Figure 1 illustrates just some of the complexities that one has to wrestle with when studying human uterine physiology whether at sub-cellular, single cell, multicellular, whole organ or whole organism levels. It is this consideration of integrated uterine function that is encapsulated by the term 'myometrial physiome'.

**Figure 2:**
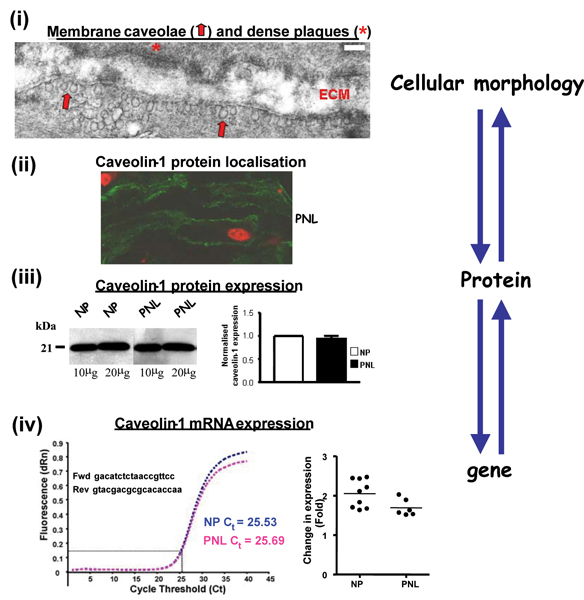
**Example of gene-protein-cellular morphology modalities for the myometrial physiome**. Investigation of the role of even one gene of the uterus – caveolin-1 in this case – crosses such modalities. **(i) **Caveolae – sarcolemmal invaginations identifiable by electron microscopy (red arrows) are thought to orchestrate uterine intracellular signalling dynamics. **(ii) **Caveolin-1 protein is an integral structural and regulatory component of caveolae. Consistent with this, caveolin-1 (green) is localised by immunofluorescent confocal microscopy primarily to the plasma membrane. **(iii) **and **(iv) **Caveolin-1 protein or mRNA expression is invariant between myometrium of non-pregnant (NP) and pregnant not in labour (PNL) women.

Underpinning this goal is our quest to answer the questions of (i) what are the cellular mechanisms regulating myometrial contractility? and (ii) what factors control the timing of onset of labour? Much of the research in the former field has been led by reductionist principles, if inadvertently. We have very focussed research questions that often allow us to assess the expression of a gene(s), the encoded protein(s) and, hopefully, make an assessment of its location and function in the myometrial cell. Determining the answer to these strictly defined questions then informs our development of further hypothesis-driven research aims. As just one example, such strategies have been employed to investigate the possible roles of proteins associated with specialised structures of the myometrial cell membrane – caveolae – in the signal processing of contractile stimuli ([6-8] and Figure 2); this, therefore, has encompassed studies of genes and encoded proteins, cell signalling dynamics, cell ultrastructure and contractile function of the myometrium. In the post-genomic age, the use of high-throughput 'omic strategies has offered alternative, but complimentary, approaches to exploring the above questions and these needn't be so deterministic in outlook [9-11]. The common link, however, is that we must take an integrative approach to interpreting any such data: for example, consider we have the question 'what are the global patterns of myometrial gene expression accompanying onset of labour?'. Collecting multiple arrays of mRNA expression data is of considerable use in answering this question yet still only tells us part of the story of how gene expression alterations contribute to uterine development and priming for parturition (term or preterm). We have to also consider how these patterns are controlled by other, epigenetic factors – *in utero *stimuli, be they endocrine, nutritional or metabolic for example, that impact upon gene expression without a change in nucleotide sequence of the genome [12]. Thus we must consider the mechanisms behind chemical modifications of DNA (such as cytosine methylation) or histones (lysine acetylation or methylation) or miRNA binding to DNA, all of which can influence gene transcript expression. Add to this cellular/regional variations in responsiveness, as well as patient biological divergence, and the complexity of analysis of even just two modalities of Figure 1 (gene expression and labour) becomes immense.

**Figure 3:**
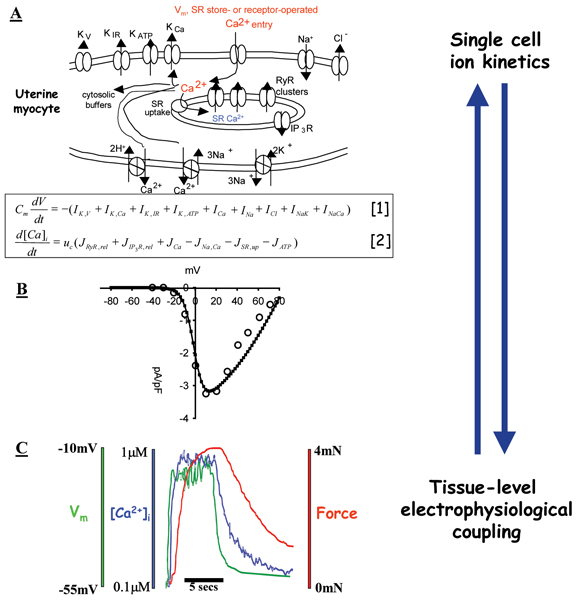
**Example of single cell to whole tissue modalities for the myometrial physiome**. **(A)** The net ionic current flowing across an uterine myocyte in unit time is the sum of many individual currents measurable by voltage clamp electrophysiology. The net current flow (equation [1]) and the cellular fluxes of intracellular Ca^2+ ^(equation [2]) can be modeled by differential equations. **(B)** Mathematical descriptions of particular ion channel characteristics can contribute to the construction of biophysically detailed models of cell excitability. Here, the inward Na^+ ^current data of [20] (open circles) can be stringently modeled as depicted by the solid line. **(C)**, this information at the single cell level must, in due course, be extended to provide a rigorous model to explain tissue level function such as that depicted here – periodic fluctuations in membrane potential, intracellular Ca^2+ ^and force occurring in situ (adapted from [21]). K_IR_, K_v_, K_ATP_, K_Ca _refer to inward rectifying, voltage-, ATP- or Ca-dependent K channels, respectively. V_m_, membrane potential. C_m_, membrane capacitance. SR, sarcoplasmic reticulum. RyR, ryanodine receptors.

**Figure 4:**
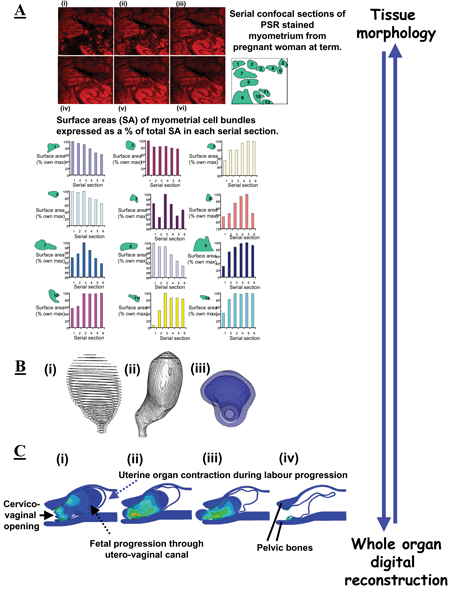
**Example of multicellular tissue to whole uterus modalities for the myometrial physiome**. Quantitative 3-dimensional reconstruction of tissue level morphological data contributes to models of whole organ function. **A**, an uterine biopsy is fixed and stained with picrosirius red and serial optical sections examined by laser confocal microscopy. The surface areas of distinct myometrial cell bundles can be quantified through the depth of the scanned tissue to monitor cellular geometry. **B**, digital reconstruction of late third trimester gravid human uterus from magnetic resonance imaging data sets; from left to right: **(i) **the 3D stacks of extracted uterine wall boundaries; **(ii) **surface view of the reconstructed 3D geometry **(iii) **semi-transparent view through the cervix. **C**, from left to right **(i-iv)**, computerised simulation of uterine and fetal movements in the final stage of active labour enables consideration of whole organ physiology in concert with pelvic skeleton biomechanics. Colour-coded brightness (blue-red) reflects intensity of pressure exerted on the fetus as it passes through the birth canal.

Computational biological methods, however, offer a promising way forward in advancing our understanding of integrated uterine function and answering the above questions. Procedures for the modelling and simulation of complex mini-system networks at all levels of modality illustrated in Figure 1 are now commonplace and a number of advanced mathematical tools can be utilised to describe genetic, metabolic, protein, signalling, single cell and multicellular networks [13]. As an example, we have begun to construct mathematical models of human myometrial function that integrate biophysical details from current experimentation and that in the literature [e.g. [14]]. Ion channel activation/inactivation kinetics data can be used to construct models of the electrical action potential of a single cell using sets of coupled differential equations (Figure 3). Similar approaches can be used to consider descriptions of the spatiotemporal flux of intracellular Ca^2+ ^(Figure 3). In concert with further experimentation – including cell-specific transcriptomics, *in vitro *and *in vivo *high resolution imaging techniques and *in vivo *pressure and electromyographic recordings [15-17] – mathematical approaches can then be utilized to gradually build the rigour and complexity of such models to consider cell-to-cell communication, tissue level cellular geometry and whole organ anatomy and biomechanics (Figure 4; [14]). Eventually, this will enable simulation of 4-dimensional patterns of uterine cellular excitability and organ contraction: a virtual physiological uterus.

## Conclusion

There is an overall goal underpinning our wish to combine theoretical and empirical approaches to understanding uterine physiology and pathophysiology. That is, to eventually establish a comprehensive and rigorous model of integrated gravid uterine function that enables the application of non-invasive *in silico *tools for predicting the physiological impact of any new treatment regimens for women at the risk of preterm labour. In other words, the creation of a virtual physiological uterus. Similar approaches have been exceptionally successful in the last 15 years at informing us of the function (and dysfunction) of isolated cardiac cells, specialised cardiac regions or the heart as a whole [18].

A final consideration concerns establishing meaningful access to the divergent datasets related to PTL for computational modelling purposes by the academic community of biologists, chemists, mathematicians and clinicians. Certainly this requires a web-based forum for the storage, and continued supplementation, of datasets in a standardized, well-defined and cross-referenced manner with open access to software tools allowing for model developments and data analysis [11,19].

## Competing interests

The authors declare that they have no competing interests.

## Authors' contributions

MJT and HZ conceived of the study, and participated in its design and coordination and helped to draft the manuscript. AB participated in the design of the study and co-ordination and, together with MJT, performed the experiments of Figure 4A. SK performed the mathematical modelling of Figure 3B. AH participated in the design of the study and contributed Figure 4B. BW contributed to the study co-ordination and Figure 4C.
